# The energy cost of action potential propagation in dopamine neurons: clues to susceptibility in Parkinson's disease

**DOI:** 10.3389/fncom.2013.00013

**Published:** 2013-03-18

**Authors:** Eleftheria K. Pissadaki, J. Paul Bolam

**Affiliations:** Medical Research Council Anatomical Neuropharmacology Unit, Department of Pharmacology and Oxford Parkinson's Disease Centre, University of OxfordOxford, UK

**Keywords:** dopamine, energy metabolism, neurodegeneration, Parkinson's disease, axons, unmyelinated, nigrostriatal pathway

## Abstract

Dopamine neurons of the substantia nigra pars compacta (SNc) are uniquely sensitive to degeneration in Parkinson's disease (PD) and its models. Although a variety of molecular characteristics have been proposed to underlie this sensitivity, one possible contributory factor is their massive, unmyelinated axonal arbor that is orders of magnitude larger than other neuronal types. We suggest that this puts them under such a high energy demand that any stressor that perturbs energy production leads to energy demand exceeding supply and subsequent cell death. One prediction of this hypothesis is that those dopamine neurons that are selectively vulnerable in PD will have a higher energy cost than those that are less vulnerable. We show here, through the use of a biology-based computational model of the axons of individual dopamine neurons, that the energy cost of axon potential propagation and recovery of the membrane potential increases with the size and complexity of the axonal arbor according to a power law. Thus SNc dopamine neurons, particularly in humans, whose axons we estimate to give rise to more than 1 million synapses and have a total length exceeding 4 m, are at a distinct disadvantage with respect to energy balance which may be a factor in their selective vulnerability in PD.

## Introduction

The selective degeneration of dopamine neurons of the substantia nigra *pars compacta* (SNc) is responsible for the principal motor symptoms of Parkinson's disease (PD). The etiopathology of their death remains unknown although many, varied and often interacting mechanisms have been proposed, including mitochondrial dysfunction, protein mishandling, inflammation, oxidative stress, genetic and environmental factors, and normal ageing (Chan et al., 2007; Sulzer, 2007; Schapira, 2008; Gasser, 2010; Guzman et al., 2010; Martin et al., 2010; Obeso et al., 2010; Surmeier et al., 2010a,b). These factors however, are not necessarily confined to SNc dopamine neurons and indeed cell death in PD is not confined to SNc dopamine neurons, but includes other classes of neurons in other regions of the brain e.g., neurons of the locus coeruleus (Gesi et al., 2000) and the pedunculopontine nucleus (Hirsch et al., 1987). Particularly vulnerable neurons are those with long, unmyelinated axons (Braak et al., 2002, 2004). Furthermore, it has been proposed that PD begins in the brain-stem and a wave of pathology progresses rostrally, when the midbrain is reached, SNc dopamine neurons degenerate and the major motor symptoms of PD ensue (Braak et al., 2002, 2004). What remains a consistent feature of idiopathic and genetic forms of PD and animal models of the disease, is the selective and exceptional vulnerability of dopamine neurons of the SNc compared to other dopamine neurons and other neurons in the brain (e.g., Hirsch et al., 1988). It has recently been proposed that a factor that contributes to their death, in addition to unique molecular characteristics (see reviews above), is the uniquely massive, unmyelinated axonal arbor, together with the hundreds of thousands of synapses established by SNc dopamine neurons which are orders of magnitude greater than other neurons (Braak et al., 2002, 2004; Wickens and Arbuthnott, 2005; Moss and Bolam, 2008, 2010; Matsuda et al., 2009; Surmeier et al., 2010a,b; Bolam and Pissadaki, 2012). The size and complexity of their axonal arbor in their principal target region, the striatum, is at least an order of magnitude greater than other classes of (less susceptible) dopamine neurons and other types of neurons in the brain (Moss and Bolam, 2010). Furthermore, extrapolation of data from rats suggests that human SNc dopamine neurons give rise to axons ten times the size and ten times the number of synapses (see “Appendix” and Bolam and Pissadaki, 2012). We propose that this axonal architecture will put SNc dopamine neurons under a high energy demand, not just to maintain normal cell biological functions, but principally in the maintenance of the resting membrane potential, the propagation of action potentials (AP) and in synaptic transmission (Harris et al., 2012). The tight energy budget that these morphological features impose on SNc dopamine neurons is likely to be a critical factor in their susceptibility in PD and animal models of PD in that they are under a bio-energetic demand that is so extreme that they are energetically “on the edge.” Under normal circumstances there is apparently little effect of this, however, any situation that perturbs the balance between energy production and demand, e.g., mitochondrial dysfunction or oxidative stress, will “tip them over the edge,” such that energy demand exceeds supply. A negative energy balance will have many detrimental consequences within a neuron including further oxidative stress, further mitochondrial dysfunction, inability to deal with protein turnover and impaired autophagy, all factors that are considered to contribute to cell death in PD (Chan et al., 2007; Sulzer, 2007; Schapira, 2008; Gasser, 2010; Guzman et al., 2010; Martin et al., 2010; Obeso et al., 2010; Surmeier et al., 2010a,b). A negative energy balance will thus lead to functional failure (die-back) and eventually, cell death (see also Wellstead and Cloutier, 2011).

A clear prediction of this hypothesis is that those dopamine neurons that are vulnerable in PD will have a higher energy demand for normal function than those that are less vulnerable. We thus sought first, to determine the energy cost of signal propagation in the axons of dopamine neurons. Although it has been suggested that energy consumption differs amongst different types of neurons (Alle et al., 2009; Sengupta et al., 2010) and is proportional to neuronal surface area (Attwell and Laughlin, 2001), the energy cost of signal propagation in large, unmyelinated, frequently branching, and highly tortuous axonal arbors is unlikely to simply relate to the total length of the axon, but will be influenced by the complexity (frequency of branching) of the arbor and is likely to be non-linear. Our approach was to create a computational model of dopamine neuron axons of different sizes and complexity which enabled us to address the issue of susceptibility as well as fundamental aspects of the neurobiology of dopamine neurons.

## Results

### Description and validation of the model neuron

**Figure 1:**
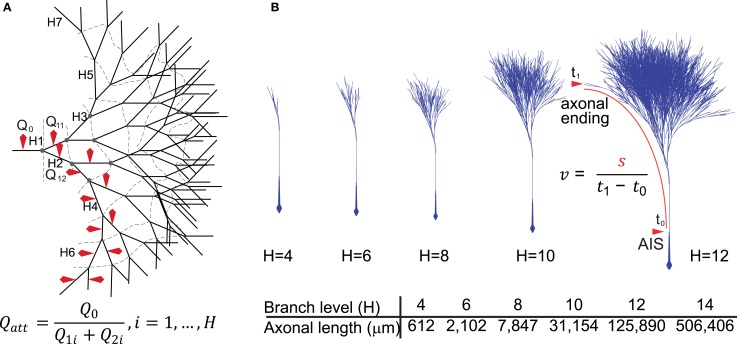
**Diagrammatic representation of the model neurons. (A)** Two-dimensional representation of the model axon based on the structure of a full binary tree. The branching level is represented by H1–7 (dashed lines represent branch contours or levels) and red markers indicate the axonal branches from where sodium charge (*Q*_*x*_) was sampled and used to derive charge attenuation (*Q*_att_) according to the formula. **(B)** A three-dimensional representation of the axonal binary tree for neurons with 4–12 levels of branching (H4–H12). For neurons with 14 levels of branches (*H* = 14), the total axonal length was about 50 cm, possessing 16,384 branches, 8191 nodes and 8192 axonal endings. We included in the models an axonal segment of ~6 mm representing the connection between the axon initial segment in the substantia nigra and the beginning of the arborization in the striatum. Conduction velocity (*v*) was calculated using the formula in which *s* represents the length of the axonal path from the axon initial segment to an axonal ending and *t*_1_ − *t*_0_ represents the time taken for the propagation of the action potential along that path.

**Figure 2:**
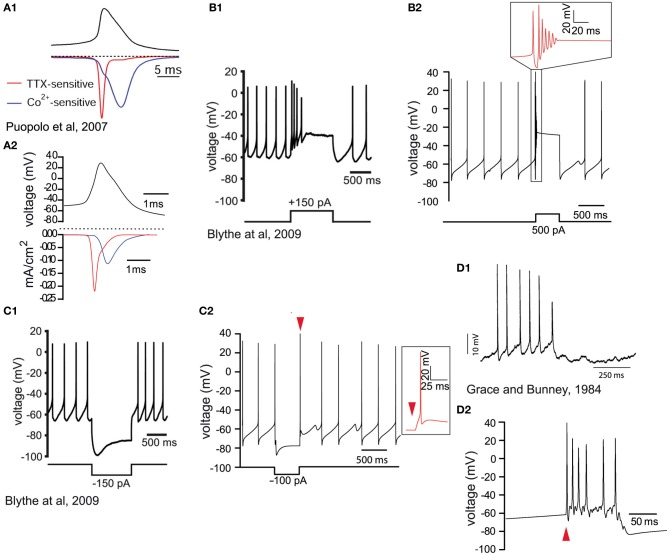
**Validation of the biophysical characteristics of the model. (A1)** Voltage waveform (black trace) during spontaneous activity of a dissociated SNc dopamine neuron. The red trace illustrates the sodium current (TTX-sensitive) and the blue trace illustrates the calcium (Co^++^-sensitive) current (reprinted from Puopolo et al., 2007). **(A2)** Voltage recording from the soma of the model (black trace). Half height action potential duration is about 1 ms. The contributions of sodium (red trace) and calcium (blue trace) currents are similar to the experimental data in **(A1)**. The longer timescale in **(A1)** is because recordings were made at 22°C and possibly a consequence of the dissociation of the neurons. **(B1)** Depolarizing current injection (lower trace) into an SNc dopamine neuron (*in vitro* slice recording) blocks spontaneous activity which then returns to baseline firing after the termination of the current injection (reprinted from Blythe et al., 2009). **(B2)** Depolarizing current injection (lower trace) in the model neuron blocks the firing of action potentials (see timescale-expanded region in the red inset) which returns to baseline firing within 500 ms. **(C1)** In response to hyperpolarizing current injection (lower trace) dopamine neurons respond with a hyperpolarization and the characteristic hyperpolarization sag mediated by *h*-current, followed by a short rebound delay (*in vitro* slice recording; reprinted from Blythe et al., 2009). **(C2)** Negative current injection into the model neuron (lower trace) leads to a similar hyperpolarization, hyperpolarization sag, and short rebound delay (see timescale-expanded region in the red inset indicated by red arrowhead). **(D1)** Intracellular recording during burst firing of a dopamine neuron *in vivo* (reprinted from Grace and Bunney, 1984a). **(D2)** Application of synaptic stimulation at the soma of the model neuron (red arrowhead) leads to burst firing with progressively reduced spike amplitude.

We implemented a compartmental biophysical model of rat dopamine neurons to estimate the energy cost linked to the propagation of an AP from its point of generation at the axon initial segment (AIS) in the SNc to the axonal endings in the striatum. Our aim was to mimic the electrophysiological profile of dopamine neurons and estimate energy cost in terms of number of ATP molecules for the restoration of ions to their resting levels after an AP has invaded the entire axonal arbor. The model was constructed in the Neuron simulation environment (Hines and Carnevale, 1997, 2001) and consists of the soma, the axon-bearing dendrite, the axon hillock, AIS, a linear segment of axon connecting the SNc to the striatum and a variable number of axonal branches within the striatum. The model was constrained by the known morphological features of rat SNc dopamine neurons obtained from individual neurons filled *in vivo* (Matsuda et al., 2009) and electron microscopic data (Moss and Bolam, 2008, 2010), including axon diameter, length, branching frequency and interbranch interval (Table 1). Beyond the AIS, the axon was composed of a 6 mm unbranched “root,” representing the projection from the SNc to the striatum, and the arborization within the striatum was represented as a full binary tree of a variable number of branches (2^height^) where height (H) represents the branch level and the number of branch points is (2^height − 1^ − 1) (Figures 1A,B). Model neurons with varying levels of axonal branching were created to represent axonal trees of SNc dopamine neurons and axonal trees of those midbrain dopamine neurons that project to the ventral striatum (see “Materials and Methods”). Thus we constructed axonal trees representing dopamine neurons that are especially susceptible in PD and those that are less susceptible, but note that we did not take into account possible electrophysiological differences between the types of neurons. The model was also equipped with passive electrical properties and active conductances to emulate the electrical activity of SNc dopamine neurons as revealed by *in vitro* and *in vivo* electrophysiological analyses (see “Materials and Methods”). These features included those that are considered to be typical of SNc dopamine neurons, such as a wide AP (Richards et al., 1997), autonomous pacemaking activity (Grace and Bunney, 1984b), depolarization block in response to positive current (Blythe et al., 2009; Tucker et al., 2012), and a hyperpolarization sag in response to negative current injection (Neuhoff et al., 2002; Figure 2, see “Materials and Methods”). At the level of the soma the model neuron had a wide AP (Figure 2A), exhibited depolarization block (Figure 2B) and reproduced autonomous activity under basal conditions and a recovery to autonomous activity after termination of injection of hyperpolarizing current (Figure 2C). Furthermore, application of synaptic stimulation to the soma led to the firing of bursts of APs (Figure 2D).

**Table 1:** **Morphological characteristics included in the model [data from Matsuda et al. (2009) and Moss and Bolam (2008, 2010)]**.

Average axonal length of DA neurons in striatum	466,800 μm
Range of axonal length of DA neurons in striatum	138,500–779,900 μm
Average inter-branch interval	31.2 ± 19.4 μm (mean ± SD)
Average fiber diameter (EM estimation)	0.34 ± 0.14 (mean ± SD)
Estimation of the number of branching points	4438–24,998
Estimation of the symmetrical synapses formed by one dopamine neurons in the striatum	100,000–250,000

**Table 2:** **Regression equations for the regression lines describing the best fit of the data points in Figures 4A–C**.

***f*(*x*) = *a* · *e*^*bx*^ + *c* · *e*^*dx*^**	***a***	***b***	***c***	***d***
ATP_Na^+^_ (neuron)	6.37 × 10^7^	−0.25	1.60 × 10^5^	0.72
ATP_Ca^++^_ (neuron)	2.61 × 10^8^	0.17	3.647 × 10^5^	0.69
***f*(*x*) = *c*· *x*^*a*^**	***c***	***a***	**R^2^/DF**	***p*-value**
ATP_Ca^++^_ (|Branches|) 20% blocked	781,628	0.968	0.9996/11	«0.0001
ATP_Ca^++^_ (Surface) 20% blocked	16,144	1.015	0.9949/11	«0.0001
ATP_Ca^++^_ (|Branches|) 40% blocked	591,562	0.959	0.9986/11	« 0.0001
ATP_Ca^++^_ (Surface) 40% blocked	13,335	1	0.9948/11	«0.0001

A double exponential (Figure 4A) and a power law function (Figures 4B,C) has been used for the regression fits.

### Signal propagation in the axonal arborization

**Figure 3:**
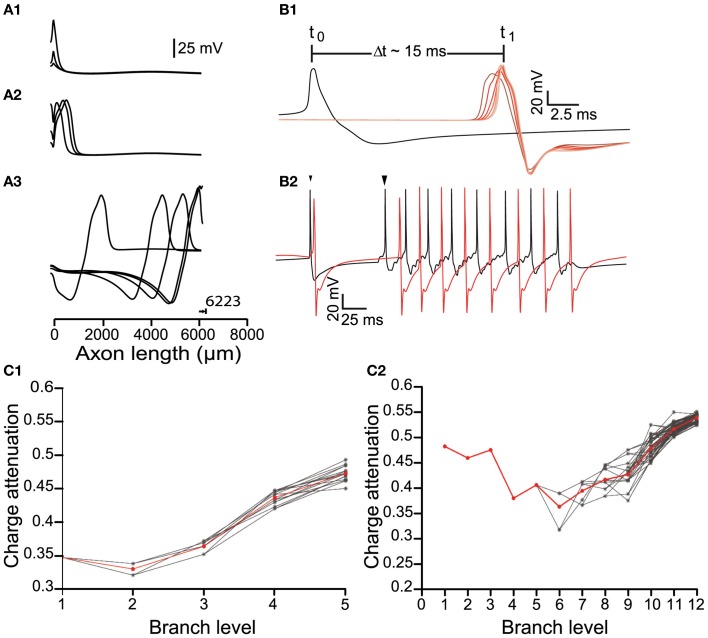
**Signal propagation throughout the axonal tree of the dopamine neuron model. (A)** Propagation of an action potential initiated at the axon initial segment (0 μm) along a path in the model neuron, represented as plots of voltage against distance. **(A1)** The action potentials reliably propagated to the axonal endings **(A2,A3)** and antidromically toward the soma. Note that 6223 μm represents the longest path through the axonal tree of a neuron with six levels of branching. **(B1)** Conductance velocity ranged from 0.45 to 0.55 m/s. For an axonal arborization with eight levels of branching, the time taken for an action potential to propagate from its point of initiation at the axon initial segment (black trace) to the axonal endings (red traces) was ~15 ms. As action potentials invaded the arborization within the striatum, their width progressively decreased and amplitude progressively increased (red traces; changing red color indicates recordings further along the axonal path). **(B2)** Synaptic stimulation facilitated action potential invasion into the higher order parts of the axonal arborization. Failure of propagation or inadequate conductance velocity of the pacemaking firing pattern (small arrowhead) was overcome by somatic synaptic stimulation (large arrowhead). Black trace: Voltage recording from the axon initial segment. Red trace: Voltage recording from an axonal ending. **(C)** The effect of branching on charge attenuation (*Q*_att_; see Figure 1A) for a small (6 levels of branching) and a large (13 levels of branching) model neuron in **(C1)** and **(C2)**, respectively. In both cases energy loss takes place to a greater degree in the distal and terminal branches of the arborization. Red trace is the mean, gray traces represent *Q*_att_ in different paths through the arborization.

We next examined how APs propagate throughout the axonal arbor in cases of different axonal length and number of levels of branching, and hence different degrees of complexity (Figure 1). In each case, the origin of the AP was the AIS and the signal orthodromically propagated toward the axonal endings (Figure 3A) and back-propagated to the soma. The model neuron matched the antidromic activation latency of ~15 ms reported by Tepper et al. (1987) by setting the conduction velocity of the signal at ~0.5 m/s (Grace and Bunney, 1980). There were changes in the shape of the AP waveform as it penetrated deeper into the axonal arborization (Figure 3B) and the propagating signal underwent an attenuation of charge in those axonal branches beyond the second half of their paths, a feature that was more evident for larger and more complex axons (Figure 3C). For axons with up to 10 levels of branching, incrementally increasing the sodium conductance (to achieve a conduction velocity of ~0.5 m/s), enabled APs generated as a consequence of autonomous activity, to invade the whole of the axonal arbor. However, for axons of greater size and complexity (>10 levels of branching), it was necessary to apply synaptic stimulation at the level of the soma to achieve successful invasion of the whole arbor because either signal propagation was not reliable or the conduction velocity was very low. This is likely to be a consequence of the requirement for greater current to charge the “membrane capacitor” of a larger axonal arbor (see “Appendix”).

### Energy cost of signal propagation

**Figure 4:**
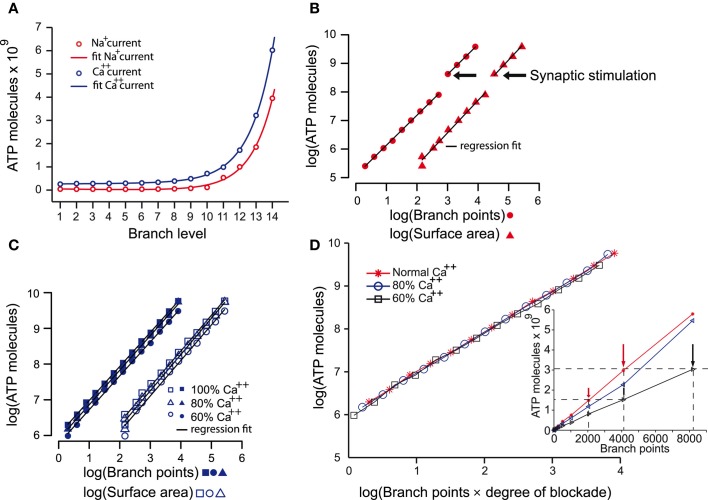
**The energy cost of the propagation of action potentials in the dopamine neuron models. (A)** Estimates of the energy cost of the propagation of action potentials throughout axonal arborizations of neurons with 1–14 levels of branching expressed as number of ATP molecules consumed. ATP molecules are consumed as a consequence of the movement of sodium ions (i.e., sodium current; red plot) and calcium ions (i.e., calcium current; blue plot). ATP demand increases exponentially with increasing levels of axonal branching for both ions (*R*^2^: Na^+^ = 0.9986; Ca^++^ = 0.9999). **(B)** A log-log plot of the number of ATP molecules consumed by the sodium current during action potential propagation against the number of branch points and the surface area of neurons with increasing levels of branching. The linear relationship of the parameters indicates that the increase in the number of ATP molecules consumed is a power law function of the number of branch points (multiple *R*^2^: Na^+^ = 0.9997, degrees of freedom = 10, *p*-value <2.2e–16) and the surface area (multiple *R*^2^: Na^+^ = 0.9965, degrees of freedom = 10, *p*-value <5.3e–16) beyond the segment connecting axon initial segment with the striatum. The discontinuity is the point at which synaptic stimulation (arrows) was required for propagation of the action potential. **(C)** Similar plot to that in **(B)** but for the calcium current. This plot shows that the increase in the number of ATP molecules consumed as a consequence of the calcium current also follows a power law relative to the two variables (except in the case of 60% Ca^++^; see Table 2). Reducing calcium conductance by 20% and 40% leads to the number of ATP molecules reducing by 10.5% and 26%, respectively (average values). The continuous lines in each plot are the regression fits for the data. **(D)** To examine how ATP is related to the reduction of the axonal network as occurs after blocking calcium channels, we plot the log of number of ATP molecules against the log of the product of number of branches and degree of calcium blockade. This reveals that the number of ATP molecules consumed is dependent on calcium and the size of the axon Blocking the calcium channels by 20% and 40% not only reduced the number of ATP molecules required for the propagation of an action potential **(C)**. The number of ATP molecules required in 60% calcium conditions for neurons with less than 8191 branch points (black arrows) was the same as for neurons one level of branching less (i.e., 4095 branch points; red arrows) in normal calcium (inset).

The creation of the model that reliably reproduced many of the characteristics of both the AP and its propagation throughout the axonal arbor of dopamine neurons put us in the position to address many questions about the fundamental biology of signal transmission in dopamine neurons (see “Discussion” and “Appendix”). The objective of the present study was to estimate the energy cost of signal propagation to assess whether the energy budget of dopamine neurons could give clues as to their susceptibility of PD (Figure 4). AP propagation in an unmyelinated axon occurs as a consequence of the influx of sodium ions through distributed voltage gated-channels and an efflux of potassium ions (Attwell and Laughlin, 2001). This, in turn, leads to an increase in the activity of Na^+^ – K^+^ ATPase pump, which acts to restore the levels of sodium and potassium ions, against concentration gradients, to resting levels. Since we know the precise density of sodium channels and the precise number of sodium ions that enter the axonal arbor for the propagation of an AP in the model, we can estimate the number of ATP molecules (and hence energy) required to operate the Na^+^ – K^+^ ATPase pump to restore the levels of ions to their resting state. Since calcium ion influx is a significant component of the axon potential in dopamine neurons, we applied the same approach for calcium. We found that the energy cost of AP propagation increased exponentially with the level of branching of the axonal arbors for both Na^+^ and Ca^++^ charge transfer (Figure 4A). To determine whether this exponential increase in energy cost is simply attributable to the monotonic increase in the number of branches and axonal surface area, we plotted the number of ATP molecules utilized to restore sodium ions and calcium ions to resting levels against the number of branch points and the surface areas (Figures 4B,C). In log-log plots the number of ATP molecules plotted against the number of branches and the surface area follow linear relationships, which implies that they are related by a power law function.

(1) $${\text{ATP}}_{{\text{Na}}^{+}}(\text{Surface})=\{\begin{array}{cc} 2692\times {\text{Surface}}^{1.137}, & \text{with synaptic stimulation}\\ 1426\times {\text{Surface}}^{1.137}, & \text{without synaptic stimulation} \end{array}$$

(2) $${\text{ATP}}_{{\text{Na}}^{+}}(|\text{Branches}|)=\{\begin{array}{cc} 288,403\times |\text{Branches}{|}^{1.051}, & \text{with synaptic stimulation}\\ 121,060\times |\text{Branches}{|}^{1.051}, & \text{without synaptic stimulation} \end{array}$$

(3) $${\text{ATP}}_{{\text{Ca}}^{++}}(\text{Surface})=23,281\times \text{Surface}$$

*R*^2^ = 0.9947/DF = 11, *p*-value « 0.0001

(4) $${\text{ATP}}_{{\text{Ca}}^{++}}(|\text{Branches}|)=1,061,696\times |\text{Branches}{|}^{0.954}$$

*R*^2^ = 0.9998/DF = 11, *p*-value « 0.0001

These findings indicate that the energy cost of AP propagation increases according to a power law function with respect to the size/complexity and/or surface area of the axonal field (Equations 1, 2). Furthermore, synaptic stimulation acts as a multiplicative index, as shown by the discontinuity of the fit (branch level 10–11) and the constant factors in the formulae. The energy cost of the restoration of calcium ions to the resting state increases according to a linear function with respect to the surface area of the neuron and to a power law function with respect to the number of axonal branches.

Finally, we examined the contribution of calcium currents to AP propagation throughout the axonal arbors. When calcium was reduced to 80% or 60%, AP propagation was successful throughout the axonal arbor for neurons of less than 10 levels of branching, but for neurons of 10 or greater levels of branching, successful propagation required synaptic stimulation. This cut-off point for the requirement for synaptic stimulation occurs at branch level 11 in “normal” calcium conditions implying that calcium currents are particularly critical deep in the axonal arbor. Interestingly, the number of ATP molecules required in 60% calcium conditions for neurons of less than 10 levels of branching (corresponding to branch points of less than 511) was the same as for neurons with one level of branching greater in normal calcium (Figure 4D, inset). This is better illustrated when the number of ATP molecules required for each case of calcium blockade are aligned against the product of number of branches and degree of calcium blockade which reveals that the number of ATP molecules is indeed dependent on calcium and the size of the axon (Figure 4D).

It should be noted that sodium conductance was reduced by about 17% and 23% in the cases of 20% and 40% reduction in calcium. Although this was not studied further, when calcium enters through voltage-gated channels, the inward calcium current contributes to depolarization of the membrane (Larkum et al., 1999; Svoboda et al., 1999) but will also lead to the activation of calcium-activated potassium channels which will lead to repolarization or spike frequency modulation (Yamada et al., 1998). Which prevails, or what the relative contribution of each, is not known for the present study but reduced requirement for sodium suggests less activation of calcium-dependent potassium channels due to reduced intracellular calcium.

### Action potential efficiency

**Figure 5:**
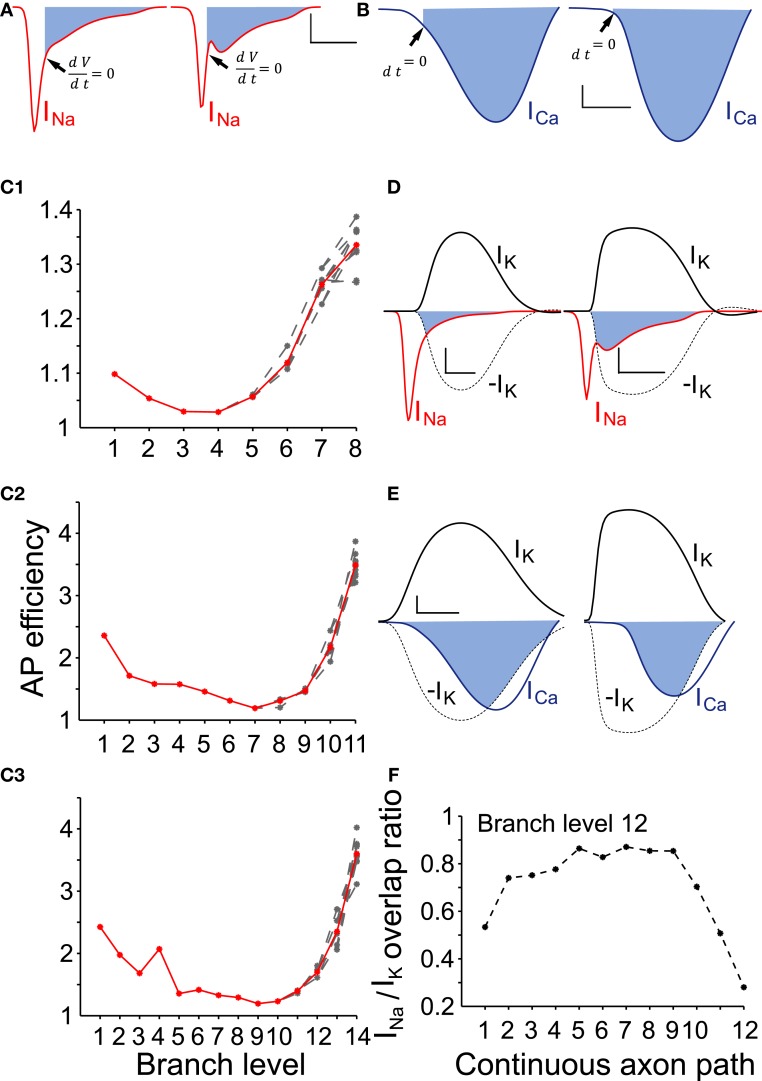
**AP efficiency depends on the neuron size and the axon branch level. (A)** To determine the action potential efficiency we calculated the ratio of Na^+^ influx integrated over the duration of an action potential to Na^+^ influx integrated over the period from the start until the peak of the action potential. Representative sodium currents recorded from a proximal (branch level = 1) and a distal axon branch (branch level = 12) during action potential propagation in an SNc model neuron with 12 levels of branching. Filled areas represent the redundant Na^+^ influx following the upstroke of the action potential. Arrows indicate the time point where *dV*/*dt* = 0, i.e., peak of the action of the action potential (scale: vertical: 0.2 mA /cm^2^; horizontal: 500 μs). **(B)** Representative calcium currents recorded from the same positions in the same model neuron. Activation of calcium channels occurs immediately preceding the peak and following the peak of the action potential (black arrows). Scale: vertical: 0.1 mA /cm^2^; horizontal: 500 μs. **(C1–C3)** AP efficiency was determined for axonal branches forming continuous paths (*n* = 10 paths) within axonal arborizations of three different sizes (8, 11, and 14 levels of branching). Action potential efficiency of more than one is indicative of energy loss (i.e., inefficiency). For all sizes of neurons, the more distal an axon-branch is located from the soma, the less energy efficient is the action potential (above a particular threshold), suggesting that the size and complexity of the axonal abrorization is negatively correlated with energy efficiency during action potential propagation. (Red curves represent the average and gray curves the individual paths) **(D)** Overlapping Na^+^ (red trace) and K^+^ (black) currents for the neuron model with 12 levels of branching. Filled areas represent charge overlap. Na^+^/K^+^ charge separation takes place to a different extent whether the axon-branch is proximal (left panel) or distal (right panel) to the axon initial segment. Dashed lines are a reflection of the potassium currents to show the regions of overlap. Scales: vertical: 0.2 mA/cm^2^; horizontal: 500 μs. **(E)** Overlapping Ca^++^ (blue trace) and K^+^ (black trace) currents at the same position as in **(D)**. Because calcium channels are mostly activated during the descending repolarization phase of the action potentials, the overlap between Ca^++^ and K^+^ currents is prominent (filled areas). Dashed lines are a reflection of the potassium currents to show the regions of overlap. Scales: vertical: 0.1 mA/cm^2^; horizontal: 500 μs. **(F)** Na^+^/K^+^ charge overlap determined in a continuous axon path of the model neuron with 12 levels of branching. Charge overlap was calculated as the ratio of the integrated non-overlapping Na^+^ charge integrated over the total Na^+^ charge during an action potential. Charge overlap ratio reaches a minimum for the axonal endings while is maximum for axon branches of moderate branch order. A ratio of less than one is indicative of less efficient action potential generation.

To determine the AP efficiency, (i.e., the efficiency of the use of sodium influx in generating the AP), we estimated the ratio of Na^+^ influx over the duration of an AP compared to the Na^+^ influx from the start until the peak of the AP (Hallermann et al., 2012). We applied this method to axonal branches of consecutive branch levels, i.e., branches belonging to a continuous path for model neurons of variable sizes. Consistent with Hallermann and colleagues, we found that branches proximal to the AIS were more efficient (less redundant sodium current influx during the down stroke of the AP) compared to the distal axonal branches (Figure 5). Furthermore, AP efficiency attained much higher values (i.e., the AP was less efficient) for model neurons of higher levels of branching (Figure 5C). We next examined the Na^+^/K^+^ charge separation as defined by the ratio of Na^+^ influx non-overlapping with K^+^ efflux over the total Na^+^ influx for a model neuron with 12 levels of branching (AP generated after synaptic stimulation). We found that the two ion fluxes were better separated for those axonal branches relatively close to the AIS (branch levels 1–8), whereas the two ion fluxes showed a greater degree of overlap in the distal axonal branches (branch levels 9–12) for a neuron model consisting of 12 levels of branching (Figure 5F). Although we have not investigated this further, it could be a consequence of uneven conduction velocity within the branched axonal arborization, which may then alter the kinetics and therefore the activation–inactivation of the local active conductances. We also measured the efficiency and degree of overlap between calcium and potassium currents during an AP. Calcium currents are activated after the peak of the AP and show a high degree of overlap with the potassium current (Figures 5B,E). Our results suggest that AP efficiency is not only location-dependent (Hallermann et al., 2012) but also dependent on the size and complexity of the axonal arborization.

## Discussion

We examined the energetic impact imposed on SNc dopamine neurons by their extensive, unmyelinated axonal arbor and asked whether the energy demand that this incurs can potentially be associated with the vulnerability of these neurons in PD. Our main findings show: (a) that the energy demand associated with AP conduction is related in a non-linear manner to the axonal size; (b) that synaptic stimulation is necessary to ensure reliable propagation throughout the axonal arbors of neurons with higher levels of branching; (c) that calcium transients facilitate signal propagation and their blockade leads to reduced signal penetration; and (d) the exponentially higher energy demands of large axons (equivalent to rat SNc neurons) compared to smaller axons (equivalent to rat VTA neurons) could underlie their selective loss in PD.

### Energy cost of action potential propagation

The model we have created suggests that the energy cost of propagation of APs throughout the axonal arbor of dopamine neurons of different sizes is defined by the size of the axonal arborization. The energy cost is not directly proportional to the surface area nor the length of the axonal arbor but rather, the energy cost increases exponentially with the number of levels of branches the axon has and grows as a power law of the size and complexity i.e., surface area and number of branch points of the axon. This disproportionally high energy demand of large axonal arbor models (equivalent to rat SNc dopamine neurons) compared to smaller axonal arbor models (equivalent to midbrain dopamine neurons projecting to the ventral striatum) may thus underlie their greater vulnerability to dopamine-selective or non-selective toxins. We calculate, based on the relatively greater expansion of the striatum (i.e., the principal target of SNc dopamine neurons) between rat and human, compared to the increase in the number of SNc dopamine neurons (see “Appendix” and Bolam and Pissadaki, 2012), that individual human SNc dopamine neurons give rise to ten times the number of synapses and ten times greater total length than rat neurons. This estimated larger size of the axon of human SNc dopamine neurons means an even greater energy cost of axon potential propagation (because of the power law relationship). We estimate, based on Equation (1), that the cost of AP propagation and recovery in a human SNc dopamine axon (17 levels of branching and about 4.14 m total length), is 9.36 × 10^10^ ATP molecules, which is an order of magnitude greater than the energetic cost for the axon of a rat SNc dopamine neuron. This higher energetic cost may thus underlie the fact that PD is a human disease; there is no known spontaneous form of PD in animals. Perhaps the negative consequences of longevity on neurons, together with the disproportionately higher energy demand of signal transmission in human SNc dopamine neurons, makes them more readily susceptible to any perturbation in energy supply which leads to an energy imbalance and subsequent die-back and cell death.

### Implications for our understanding and treatment of PD

If indeed, energy demand (and/or perturbation of energy supply) is a major factor in dopamine neuron death then our findings suggest that PD is not a specific disease with a unique molecular trigger, rather, they suggest that PD is a “general” disease in that many factors that act as stressors of neurons, may contribute to the energy imbalance and neurodegeneration. This may offer an explanation for the wide variety of proposed molecular triggers of PD, the wide variety of gene products associated with genetic forms of PD and the many proposed pathways to cell death (see “Introduction” for references): all contribute to the perturbation of the cell biology of (possibly all) neurons, but selective loss of dopamine neurons is a consequence of their unique structure and consequent high energy budget. Whatever the situation, our study emphasizes the point that it is critical to consider not only the cell body and dendrites of dopamine neurons in analyses of their function and dysfunction, but also their axonal arbor which accounts for a high proportion of the surface area and volume of dopamine neurons. Do these findings give clues as to how to treat PD? One may speculate that manipulation of mitochondrial biochemistry or genetic manipulation of dopamine neurons so as to enhance energy production would be protective and beneficial. Of course, as it is recognized that the principal motor symptoms of PD do not emerge until there is about a 60% loss in dopamine and dopamine neurons, these potential avenues, as is the case for all neuroprotective therapies, depend on the early, pre-symptomatic diagnosis of PD.

### Implications for our understanding of the fundamental neurobiology of dopamine neurons

We created the model of the axons of dopamine neurons to test a hypothesis about the energy cost of their large and complex axonal architecture and indeed, the model enabled us to address the hypothesis. However, a computational model of a neuron is likely to enable us to address other properties of the neuron in question. This is the subject of on-going work. However, the model has already raised several issues about the neurobiology of dopamine neurons.

#### Action potential firing and the invasion of the axonal tree

The dopamine neuron model exhibited autonomous firing of APs that invaded the whole axonal arbor in the smaller neurons (≤9 levels of branching). However, in the larger neurons (>9 levels of branching), this was not the case; synaptic stimulation was required for APs to invade the whole of the axonal arbor (see “Appendix”). If this finding translates to real dopamine neurons *in vivo* then there are important implications concerning the basal release of dopamine at the level of the striatum, which is assumed to arise as a consequence of autonomous firing. It would suggest that under resting conditions, dopamine release occurs throughout the axonal arbor in smaller dopamine neurons but only part of the axonal arbor of larger dopamine neurons. Sub-types of dopamine neurons with different dopamine release characteristics depending of the size and complexity of the axonal arbor, could decrease the redundancy in the ascending dopaminergic system and increase task selectivity. The variability in the size of the axonal arbors of labeled dopamine neurons in the SNc described by Matsuda et al. (2009) are consistent with this idea.

#### Action potential efficiency

Our study suggests that AP conduction in SNc dopamine axons is inefficient, i.e., it involves excess Na^+^ flow, and the degree of inefficiency grows with the number of levels of branches that a neuron possesses. It has been suggested previously that excess Na^+^ influx takes place in fast spiking neurons and has been proposed as a mechanism to promote reliable propagation at high frequencies whereas fast inactivation of Na^+^ channels do not seem to promote propagation (Hallermann et al., 2012). Whether dopamine neurons support high frequency conduction throughout their axonal arbor is not known, however, it is evident that high frequency burst do lead to dopamine release in the striatum (Floresco et al., 2003). In this respect, as predicted by the neuron model, excess Na^+^ influx during burst activity is likely to support conduction in the largest axons. At the same time the excessive amount of intracellular sodium at the distal axon branches may accumulate and impose an extra energetic load to the Na^+^/K^+^-ATPase. As the Na^+^/K^+^-ATPase pump is electrogenic, the outward, positive ionic current dealing with excess Na^+^, may hyperpolarize the membrane and impair synaptic efficacy and transmission (Johnson et al., 1992; Shen and Johnson, 1998). Furthermore, inadequate Na^+^ extrusion, may decrease the Na^+^ gradient, increase the intracellular sodium concentration and lead to dysfunction of the Ca^++^/Na^+^ exchanger (Blaustein and Lederer, 1999). Both of these paths could contribute to the loss of striatal terminals occurring in animal models of PD and which precedes the progressive degeneration of dopamine neuron somata in the SNc (Meredith et al., 2008).

#### Role of calcium conductance

L-type calcium currents are important for the pacemaking activity of the SN neurons (Nedergaard et al., 1993; Amini et al., 1999; Wilson and Callaway, 2000; see also Guzman et al., 2009) and it has been proposed that Ca^++^ entry drives the mitochondrial stress related to PD and neurodegeneration (Surmeier et al., 2010a,b). Our findings concur in that they suggest that calcium conductance is involved in the conduction of APs in dopamine neurons. We show that Ca^++^ currents are more energy expensive than Na^+^ currents, partly because the plasma membrane calcium-ATPase extrudes one calcium ion at the cost of one ATP molecule. Thus calcium contributes significantly to the overall energy budget of the neuron and this may impose stress on those mechanisms that support calcium dynamics. Such mechanisms could be the endogenous low buffering capacity of SN dopamine neurons (compared to VTA neurons; see also Foehring et al., 2009), the calcium-ATPase, the Na^+^/Ca^++^ transpose system and even the mitochondrial Ca^++^ buffering. Any stress on these mechanisms can alter the calcium homeostasis and may contribute to toxic damage.

Calcium transients have a major impact when they are present in the dendritic compartments of a neuron; by depolarizing the membrane, they actively promote the propagation of APs and of EPSPs (Johnston et al., 1996a). We show here that besides the considerable energetic cost of calcium currents, their presence augments AP invasion to the very distal axonal terminals. The likely clustering of calcium channels at axonal varicosities *in vivo* (Cox et al., 2000) may suggest that an additional function of varicosities is the promotion of the invasion APs into the full axonal arbor.

#### Oscillations in the axon

The model also gives clues about the oscillatory nature of dopamine neurons (see “Appendix”). Our results are suggestive that the axons of the SNc dopamine neurons may serve as a third physical site, in addition to the soma and the dendrites (Wilson and Callaway, 2000), for generating oscillatory behavior. If this is the case, then it would significantly add to the energy budget of individual SNc dopamine neurons as it is likely to oscillate at its own natural frequency and because of its size, it will far outweigh cost of oscillatory activity of the somatodendritic domain.

### Limitations of the model

The major limitations of our model arise from the lack of empirical data relating to the types, distribution and properties of channels present in SNc dopamine neuron axons. A number of studies have shown that sodium channels in the axonal membrane may differ from (Martina and Jonas, 1997), and may have faster activation and inactivation kinetics (Engel and Jonas, 2005; Schmidt-Hieber and Bischofberger, 2010) than, somatic channels which would result in less current flow (Schmidt-Hieber and Bischofberger, 2010). On the other hand, it has been shown that sodium efficiency i.e., the energy cost of sodium channel conductance, is more dependent on the shape of the AP and less on the sodium channel kinetics at the soma (Carter and Bean, 2009). Our findings suggest that the inclusion of SNc-specific axon conductances, when the data become available, will only quantitatively change the results, because the factors dictating energy cost are mainly governed by the geometry of the axonal structure. Furthermore, potassium channels, especially those with fast kinetics, are associated with narrow APs, increased sodium influx and thus, have a high energy cost (Carter and Bean, 2009; Hasenstaub et al., 2010). In the absence of more precise data, we have been conservative and inserted the minimum of fast potassium channels, so as not to mediate fast spiking behavior which is not a characteristic of SNc dopamine neurons.

APs involve the influx of calcium ions in mammalian axons. The model neuron encompasses those calcium channels known to mediate striatal dopamine release such as N-, R-, T-, P-, and L-type calcium channels. Peak conductance is hierarchically set (Weber et al., 2010) however, they are not SNc dopamine neuron-specific.

Continuous conduction in unmyelinated axons is mediated by uniformly distributed ion channels along the axon membrane (Attwell and Laughlin, 2001) and indeed, in our model sodium and potassium channels were distributed uniformly. However, it should be noted that it has been reported in invertebrates, that clustering of potassium and sodium channels may occur in the unmyelinated axolemma and can mediate continuous signal propagation (Johnston et al., 1996b). Clustering of potassium channels has been shown to increase propagation efficiency and conduction velocity of APs, whereas clustering of sodium channels is beneficial under a particular range of axonal properties (Zeng and Tang, 2009). The issue of the clustering of channels will be explored in future work. Furthermore, we have not included an ATP pump in the model neuron. Because an activated pump is electrogenic, it is expected that its inclusion would affect the hyperpolarization phase following an AP. This also will be explored in future work.

The model neuron had been calibrated previously with different sets of parameters relating to the conductances associated with the calcium, sodium, potassium and h channels (Pissadaki and Bolam, 2010). This led to different values for ATP consumption, however, it did not lead to qualitatively different results. Additionally, the axonal arborizations had been initially implemented using a thicker axon branch diameter (0.82 ± 0.11 μm) (Matsuda et al., 2009) than those used in the present study (0.34 ± 0.14 μm). A prominent difference introduced by this structural change was the reduced ability of the signals to penetrate the full axonal arbor; only by applying synaptic stimulation in larger neurons (but at an earlier level than with the smaller diameters used in the current study) were signals reliably propagated throughout the axonal tree.

It should be noted that the calculations that we have made only address the costs of AP propagation and recovery of the membrane potential. Dopamine neurons, particularly those of the SNc will also have a large energy demand for neuron-specific activities like vesicular release of transmitter, transporters and, in addition, normal cell biology functions which may exacerbate the situation.

## Conclusions

Our results suggest that the unique axonal architecture of rat SNc dopamine neurons puts them under a disproportionately high energy budget for signal transmission. Extrapolation of these findings to human SNc dopamine neurons suggests that they are under an even higher energy budget. We suggest that energy supply/demand may play a critical factor in dopamine neuron death and may be a common pathway for the many factors that contribute to the neurodegenerative process in PD.

## Materials and methods

The SN dopamine neuron model consists of the soma, the axon-bearing dendrite, the axon hillock, the AIS and a variable number of axon branches. Constraints on the model were derived from data relating to the shape and neuroarchitecture of the SNc dopamine neurons principally from the work of Matsuda et al. (2009) and Moss and Bolam (2008, 2010) and data relating to the *in vivo* and *in vitro* electrophysiological properties of dopamine neurons. The number of segments for each section were determined from the d-lambda rule (Hines and Carnevale, 2001). The backward Euler integration was used for all the simulations with constant time step of 25 μs. Data analysis was performed using MATLAB (Mathworks, Inc) and the R-project for Statistical Computing. The model will be available from: http://senselab.med.yale.edu/ModelDB/.

### Neuron model geometry

The neuronal structure was synthetically constructed in the Neuron Simulation Environment (Hines and Carnevale, 1997). The arborization of the axon was implemented as a full binary tree of variable height (height, H, of a full binary tree is the number of levels within the tree, referred to as branch level) in two dimensions using the following algorithm:

$$\begin{array}{l} \text{axon}(2k+1)\text{connect to axon}(k),\\ \text{axon}(2k+2)\text{connect to axon}(k),k=0,\ldots ,\frac{\text{axonNumber}}{2}-1 \end{array}$$

where axonNumber = 2^height^ − 1

It was then converted to 3D geometry according to the following procedure:

(5) $$x_{n+1}=x_{n}+r\sin(a_{1})\cos(a_{2})$$

(6) $$y_{n+1}=y_{n}+r\sin(a_{1})\sin(a_{2})$$

(7) $$z_{n+1}=z_{n}+r\text{cos}(a_{1})$$

(8) $$a_{1}=\text{arccos}((z_{n}-z_{n\;-\;1})/r)$$

(9) $$a_{2}=\text{arctan}((y_{n}-y_{n-1})/(x_{2}-x_{1}))$$

(10) $$r=\sqrt{{\left(x_{n}-x_{n-1}\right)}^{2}+{\left(y_{n}-y_{n-1}\right)}^{2}+{\left(z_{n}-z_{n-1}\right)}^{2}}$$

**Figure A1:**
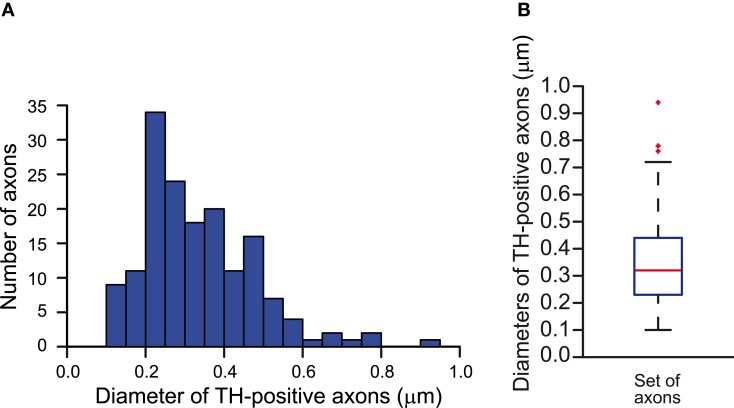
**Estimation of the diameters of the axons of SNc dopamine neurons in the striatum of the rat. (A)** Frequency distribution of the diameters of tyrosine hydroxylase-immunopositive axons measured in electron micrographs of the striatum (*n* = 161). **(B)** Whisker plot of the distribution of the diameters of tyrosine hydroxylase-positive axons. Median diameter = 0.32 μm. Red markers indicate outlier data, not included in the calculations of the minimum and maximum whiskers.

The mean interval (mean ± SD) between two axonal branch points, namely the interbranch interval, was (31 ± rand_1_ μm) and the diameter of the axon was set at (0.34 ± rand_2_ μm), where rand_1_ and rand_2_ are uniform random variables over the interval (−19, 19) and (−0.14, 0.14), respectively. The diameter was obtained by the analysis of tyrosine hydroxylase-immunolabeled (i.e., dopaminergic) axons in electron micrographs of rat striatum. The micrographs were part of the data-set used in the analyses of Moss and Bolam (2008) (Figure A1). No tapering of the axon was applied.

Different sizes of axonal arborizations were obtained by the inclusion of different numbers of axon segments and different numbers of bifurcations. The maximum diameter of the soma was set at 30 μm and its total surface as 854 μm^2^ (Tepper et al., 1987). The axon-bearing dendrite was directly attached to the soma and its diameter tapered reciprocally with the distance from the soma, ranging from 6 to 3.2 μm. It was then connected to the axon hillock, the diameter of which, tapered from 3.8 to 1.7 μm. The 1.7 μm thick starting point of the AIS was connected to the axon hillock (1.2 μm diameter) and then to the first axon segment (1.0 μm diameter). The diameter of the AIS was set to be positively correlated with the maximum diameter of the somatic region (Sloper and Powell, 1979). The first axon segment was the “root” of the binary tree, i.e., the segment connecting the SNc to the striatum and a binary axon tree constructed from that point onwards i.e., the point of arrival in the striatum. The diameter of the root (mother branch) was set according to Equation (11) after the diameters of the first two daughter branches were randomly selected. With this configuration we ensured that the geometrical ratio (Goldstein and Rall, 1974) (GR) was equal to 1 (using the equation below):

(11) $$\text{GR}=(d_{1}^{3/2}+d_{2}^{3/2})/d_{\text{mother}}^{3/2}$$

### Electrophysiological properties of the model neuron

#### Passive properties

Passive membrane parameters of the model were assigned as follows: membrane capacitance (C_m_) 1 μF/cm^2^, membrane resistance for the soma and dendrites was 60 KΩ-cm^2^ and axial resistance was 250 Ω-cm. Membrane resistance for the axon segments and the AIS was 150 KΩ-cm^2^ and axial resistance was 70 Ω-cm. Values are comparable to Hausser et al. (1995); Alle et al. (2009); Mainen et al. (1995) and Hu et al. (2009). Membrane potential at rest was equal to −56 mV (Grace and Bunney, 1984b).

#### Active properties

The model neuron was endowed with a variety of active conductances known to occur in SNc dopamine neuron somata. Channel mechanisms inserted in the model were used with little or no modification from other published compartmental models (Chan et al., 2007; Mercer et al., 2007; Deister et al., 2009; Hu et al., 2009; Pissadaki et al., 2010). Densities and distributions of these mechanisms are unknown for the axonal and dendritic sections. We assumed uniform sodium channel density for soma and dendritic membrane (see Table A1). The AIS was equipped with a smaller number of active conductances and a high, ramp-shaped increase/decrease of Na_v_1.6 sodium conductance resembling data from Schmidt-Hieber and Bischofberger (2010), with a maximum value of 3000 pS/μm^2^, intermediate value of 1500 pS/μm^2^ and minimum value of 700 pS/μm^2^. As little is known about the active conductances in the axons of SNc dopamine neurons, empirical data available from other brain neurons served as guidelines. The axon segments were endowed with Na_v_1.6 and Na_v_1.2 sodium channels and additional types of active conductances including several types of potassium channels and subtypes of voltage-gated calcium channels (VGCC) (Table A1).

#### Signal propagation: sodium channels

Our objective was to construct and validate a biophysical model of the SNc dopamine neurons in order to estimate the energy cost of the propagation of APs. One of the most critical questions of this study was whether APs reliably invade every branch of the long and tortuous axon arborization that dopamine neurons possess. Failure of propagation has been reported in invertebrates and in a number of mammalian systems (Foust et al., 2010). Propagation failure takes different forms and is mostly dependent on the frequency of stimulation, on the embedded irregularities of an axon such as branching points, on the geometrical ratio of mother and daughter diameters, and on varicosities and swelling perturbations (Debanne, 2004), all of which may add an electrical load to the invading AP (Goldstein and Rall, 1974). Branch points in spinal cord neurons may boost AP propagation by the expression of a high density of sodium channels (Engel and Jonas, 2005). Purkinje neurons reliably propagate simple APs into axon collaterals whereas spikelets of complex spikes have variable success in propagation (Cox et al., 2000; Foust et al., 2010; Palmer et al., 2010). Furthermore, APs of neocortical pyramidal neurons reliably invade electrotonically distant regions of a branching axon (Cox et al., 2000) and densely varicose and branched Schaffer-type unmyelinated axons reliably conduct AP of high and low frequencies (Raastad and Shepherd, 2003). In the present study we thus assumed that APs are reliably propagated throughout the axonal arborizations.

#### Conduction velocity

**Figure A2:**
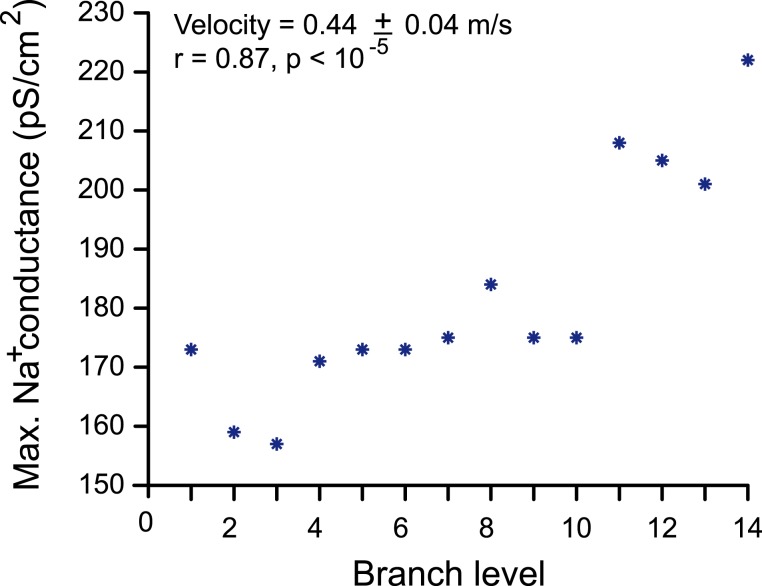
**Relationship between sodium channel conductance and level of branching.** Sodium channel conductance and level of branching of a neuron are positively correlated when signal propagation was constrained by the maximum conduction velocity (0.5 m/s) and the action potential magnitude exceeding membrane potential of 0 mV.

A critical component for ensuring reliable signal propagation is the density and distribution of sodium channels (Huxley, 2002). The density of sodium channels is necessarily greater in unmyelinated axons as propagation is continuous as opposed to saltatory in myelinated axons (Debanne, 2004; Debanne et al., 2011). To ensure reliable propagation throughout the axonal arborizations we progressively increased (multiplication steps of 1.02 or 1.01) the sodium channel density at the axon branches (except model neurons with 1 or 2 levels of branching). This condition was limited by the conduction velocity of 0.5 m/s for SNc dopamine neurons measured *in vivo* (Grace and Bunney, 1980); the channel density was adjusted to achieve this conduction velocity. Furthermore, an AP that exceeds 0 mV membrane potential together with the conduction velocity of 0.5 m/s was considered as successful signal propagation. Sodium channels, as well as the rest of the inserted mechanisms (excluding calcium channels), were evenly distributed in the axons. Figure A2 depicts the incremental increase of sodium density relative to the level of branching of the cell.

#### Calcium channel density and distribution

Striatal dopamine release has been shown to involve N-type P/Q-type, L-type, and R- and T-type calcium channels (Okita et al., 2000; Chen et al., 2006). While the importance of calcium transients induced by signal invasion in dopamine release is clear, the precise number of calcium ions and the specific channel types involved during a single or a burst of APs is unknown for the SNc dopamine axons and the embedded varicosities. To resolve this issue we adopted the conductance hierarchy Cav2.2 > Cav1 > Cav3 reported by (Weber et al., 2010). In addition, VGCC have been shown to be concentrated in varicosities (Cox et al., 2000) and varicosities are likely to be the sites of dopaminergic transmission. The high density of varicosities [1 varicosity per μm proposed by Arbuthnott and Wickens (2007), we used 0.8 varicosities per μm^2^] suggests the presence of hot spots and clustered calcium mechanisms. Because of the high density of varicosities, adding extra spatial resolution to the model would not substantially alter the final picture. Instead, hot spots were modeled according to the following:

(conductance density in the axon branch containing the hot spot)×(area of the segment)=(hot spot conductance density)×(hot spot area).

Several studies have shown that synaptic vesicle fusion can be gated by a single calcium channel nanodomain (Weber et al., 2010). If the single-channel conductance for the N-type calcium channel is 2.8 pS (Weber et al., 2010) and the number of fused vesicles per AP invasion is ten, this makes a peak conductance of 0.00224 S/cm^2^. Accordingly, L-type calcium channels with a single-channel conductance of 2.2 pS (Weber et al., 2010) will end up with a peak conductance of 0.00174 S/cm^2^. T-type calcium peak conductance was set at 2.85 × 10^−5^ S/cm^2^. We explored the consequences of reduced calcium transients by blocking axon-inserted calcium conductances by 20% and 40%. In setting the calcium channel conductance with this scheme we aimed to establish a realistic representation of the calcium transients that would eventually lead to reliable predictions.

### Validation studies (Figure 2)

In creating a computational model it is critical to ensure that the model neuron emulates the electrophysiological profile of the neuron under study. We set the soma as a reference point and we calibrated parameters according to published data. Distinct features of the electrophysiological activity of SNc dopamine neurons that we include are: the generation of broad APs (Grace and Bunney, 1984b; Bean, 2007), the large sag component in response to hyperpolarizing current injection (Neuhoff et al., 2002; Blythe et al., 2009), depolarization block following depolarizing current injection (Blythe et al., 2009), and a large calcium current activated during the repolarization phase of AP (Puopolo et al., 2007). The validation was performed in a reduced version of the model neuron consisting of only the soma, the axon bearing dendrite, the axon hillock, the AIS and a single axonal branch.

#### The action potential

SNc dopamine neurons have long duration APs (1.49 ± 0.03 ms) with large amplitude after-hyperpolarization potentials (AHP) (Richards et al., 1997). The major ionic conductances of an AP are the tetrodotoxin-sensitive sodium current during the upstroke and the cobalt-sensitive calcium current during the downstroke (Puopolo et al., 2007). Peak absolute sodium current at the soma of the model neuron was ~0.2 mA/cm^2^ versus 0.14 mA/cm^2^ for a somatic surface of 707 μm^2^ (Puopolo et al., 2007). Absolute calcium channel current peak was at ~0.1 mA/cm^2^ compared to 0.11 mA/cm^2^ provided from experimental data (Puopolo et al., 2007). By integrating the charge over the time window of an AP, we obtain the charge entering the neuron carried by each of the two ionic mechanisms. Sodium charge entering the soma during a single AP for the model neuron is 992 fC and 1240 fC for sodium and calcium currents, respectively. Our values are different from the values provided by Puopolo et al. (2007), (1671 ± 151 fC and 3534 ± 744 fC for sodium and calcium currents, respectively). This is most probably due to the different duration of APs between our model and the reported study which was carried out at 22°C. Values have been obtained by integrating the charges over the time window of an AP at the soma region.

#### Burst activity induced by synaptic stimulation

**Figure A3:**
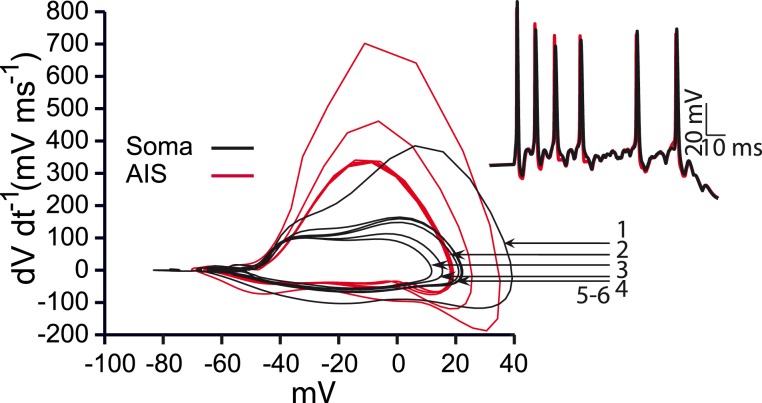
**Phase plots during bursting activity.** Red curves represent activity recorded from the action initial segment and black curves represent activity recorded from the soma. The first three spikes have successively reduced amplitudes and the rate of voltage change drops indicating smaller total amounts of inflowing current. Arrows indicate the number of the somatic spike in the burst depicted in the inset.

Synaptic stimulation (mediated by AMPA, NMDA, GABA_A_, and GABA_B_ simulated currents) at the level of the soma led the model neuron to discharge with bursts followed by a prominent hyperpolarization phase and recovery of the autonomous firing after ~3 s (Grace and Bunney, 1984a; Nedergaard, 2004). AP amplitude reduced and interspike interval increased as the burst progressed, this was particularly prominent for the first few spikes of a burst (Figure A3).

### Methods for estimation of energy costs (Figure 3)

#### Estimation of energy cost of action potential propagation

**Figure A4:**
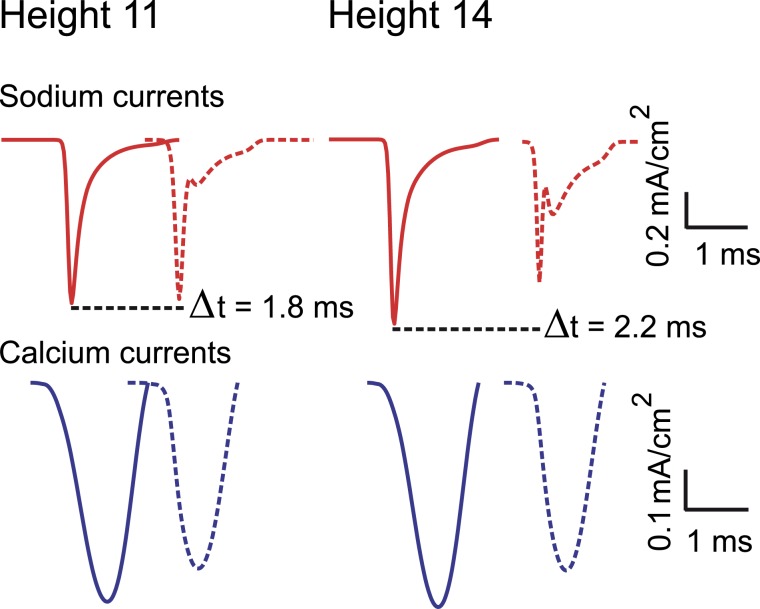
**Sodium (upper panel) and calcium (lower panel) currents composing an action potential (AP).** Sodium and calcium currents recorded during the propagation of an AP in a continuous path of axonal arborizations of neurons with 11 and 14 levels of branching. The first curve of each panel was recorded from the first axonal branch of the path and the second (dashed curve) from the last. Peak amplitude currents attenuate as they regenerate at the axonal endings. Also evident from the plots is the propagation latency within the axonal path.

Influx of sodium ions during the conduction of APs increases the activity of the Na^+^-K^+^- adenosine triphosphatase (ATPase) to restore the intracellular/extracellular concentrations of sodium and potassium ions; the estimation of charge transferred for the generation and propagation of AP is thus proportional to energy consumption. APs in dopamine neurons also involve the influx of Ca^++^ down a concentration gradient through VGCCs. The influx is balanced by the uphill movement of calcium ions mediated by the Ca^++^-ATPase pump or the sodium-calcium exchange, in an energy-dependent manner (Aisley, 2001). For the purposes of the model we have used a mechanism mediating the process of the calcium pump, calcium diffusion and calcium buffering (Hines et al., 2004), inserted uniformly throughout the somatodendritic and axonal architecture. Estimations of the energy expense of calcium influx are based on the total calcium current entering the cell. To estimate the energy demand for the propagation of a single AP, generated at the AIS, to the axonal endings in the striatum, we extracted the sodium and calcium branch currents recorded from a path of the axonal tree (Figure A4). Knowing the current density of Na^+^ and Ca^++^, the transferred charge was estimated from the time integral of the two major currents driving the AP (Equations 12–14).

To estimate the number of ions entering during an AP, we divided the transferred charge by the electric charge, *e* (2^*^e for the calcium charge). To obtain the number of the ATP molecules, we divided by 3 and by 1 for the number of sodium and calcium ions, respectively Equations (13, 14). Finally, we multiplied the number of ATP molecules (per branch and per level) with the surface area of the branches at each level.

(12) $$Q_{\text{Na}}=\int I_{{\text{Na}}^{+}}dt,\;Q_{\text{Ca}}=\int I_{{\text{Ca}}^{++}}dt$$

(13) $$\left|{\text{Ions}}_{{\text{Na}}^{+}}\right|=Q_{\text{Na}}/e\to \left|\text{ATPmolecules}\right|=\left|{\text{Ions}}_{{\text{Na}}^{+}}/3\right|$$

(14) $$\left|{\text{Ions}}_{{\text{Ca}}^{++}}\right|=Q_{\text{Ca}}/2e\to \left|\text{ATPmolecules}\right|=\left|{\text{Ions}}_{{\text{Ca}}^{\text{++}}}\right|$$

### Conflict of interest statement

The authors declare that the research was conducted in the absence of any commercial or financial relationships that could be construed as a potential conflict of interest.

## Appendix

### Estimation of numbers of synapses and the sizes of dopamine neurons axons

From knowledge of the number of dopamine neurons in rat SNc (12,000; Nair-Roberts et al., 2008) and the volume of striatum (19.9 mm^3^; Oorschot, 1996) we calculate there is one SNc dopamine neuron per 0.001658 mm^3^ of striatum. On the basis of the synaptic organization of the striatum recalculated from the data of Moss and Bolam (2010), we estimate that a single SNc dopamine neuron gives rise to 102,081–244,995 synapses at the level of the striatum (the range is due to different estimates of the number of spines in the striatum) and according to Matsuda et al. (2009), have a mean total length of 46.7 cm (range 13.8–77.9 cm). A larger number of midbrain dopamine neurons in the ventral tegmental area (~20,000; Nair-Roberts et al., 2008) innervate a volume of forebrain (ventral striatum) of about one fifth of the volume of the dorsal striatum (~4.0 mm^3^); there is thus one dopamine neuron per 0.0002 mm^3^ of ventral striatum. Assuming the synaptic organization is the same, then we can estimate that an individual dopamine neuron innervating ventral striatum gives rise to 12,351–29,644 synapses (number synapses for SNc neuron × ratio of volume of ventral/dorsal forebrain innervated per dopamine neuron) with an average total length of 5.64 cm (i.e., 0.121 × 46.7 cm). In humans the volume of striatum (6280 mm^3^; Yin et al., 2009) has increased ten times more than the increase in number of dopamine neurons in the SNc (382,000; Hardman et al., 2002) compared to rat. So the volume of striatum per SNc dopamine neuron in humans is 0.01644 mm^3^ i.e., 9.9 times that in the rat. Again, if we assume the principles of synaptic organization in human are the same as in rat, then we estimate that SNc dopamine neurons in humans gives rise to 1,010,601–2,425,450 synapses, with an average total length of about 4.6 m. For more detailed discussion see Bolam and Pissadaki (2012).

**Table A1:** **Channels and conductances inserted in the model**.

**pS/um^2^**	**Soma, dendrites hillock**	**AIS**	**Axon branches**
$\bar{g_{\text{Na}}}$	720 (^*^)	–	–
$\bar{g_{\text{Nav1}.\text{6}}}$ (Hu et al., 2009)	–	700–3000	from 170
$\bar{g_{\text{Nav1}.\text{2}}}$ (Hu et al., 2009)			0.1512
$\bar{g_{\text{Kv2}}}$ (Mercer et al., 2007)	54	3600	630
$\bar{g_{\text{Kv1}}}$ (Mercer et al., 2007)	5	8	1
$\bar{g_{\text{Kv4}}}$ (Mercer et al., 2007)	12.6	8	5.6
$\bar{g_{\text{Kcnq}}}$ (Chan et al., 2007)	1.8	–	–
$\bar{g_{\text{SK}}}$ (Chan et al., 2007)	2.05	1.026	1.026
$\bar{g_{\text{HCN}}}$ (Wang et al., 2002)	0.9	2.7	0.3
$\bar{g_{\text{CaN}}}$ (Pissadaki et al., 2010)	0.1	–	22.4
$\bar{g_{\text{fAHP}}}$ (Shao et al., 1999)	27.5	25	25
$\bar{g_{\text{Nap}}}$ (Pissadaki et al., 2010)	10.4	10.4	8
$\bar{g_{\text{CaP}}}$ (Chan et al., 2007)	0.15	0.15	–
$\bar{g_{\text{CaL}}}$ (Chan et al., 2007)	10	10	0.285
$\bar{p_{\text{CaR}}}$ (Wolf et al., 2005)	0.52	0.52	–
$\bar{g_{\text{CaT}}}$ (Wolf et al., 2005)	1.14	1.14	17.4
$\bar{p_{\text{CaQ}}}$ (Steephen and Manchanda, 2009)	10	10	–

* The Nav1.2 type of sodium channel (modified with V_half_activation_ = −15.6 mV).

### Predictions of the model relating to the neurobiology of dopamine neurons

#### Axons may be the third generator of oscillatory activity in dopamine neurons

It has been suggested that the SNc dopamine neurons can be modeled as a set of electrically coupled oscillators consisting of the soma and the dendrites (Wilson and Callaway, 2000). To assess the possibility that the axon may act as an oscillator, we set the resting membrane potential throughout the neuron model, except the axonal arborization, to a non-negative value (0 mV). In this situation autonomous activity was initiated at the level beyond the AIS. Although this observation requires further examination, it raises the possibility that the axon of SNc dopamine neurons is a potential site for generation of, and sustaining, autonomous activity.

#### The necessity for the synaptic stimulation for action potential propagation

In a simplified, single compartment neuron model with only the sodium conductance present, the membrane current (*I*_*m*_) equals to *I*_*m*_ = *I*_*C*_ + *I*_*i*_, where I_C_ is the capacitor current and I_i_ is the ionic (sodium here) current. Similarly,

(A1) $$I_{m}=C\frac{dV}{dt}+(V-E_{\text{Na}})\cdot g_{\text{Na}}$$

Since the ionic current supplies current to the capacitor then *I*_*C* = −*I*_*i*__ and Equation (A1) is then equivalent to:

(A2) $$C\frac{dV}{dt}=(E_{\text{Na}}-V)\cdot g_{\text{Na}}$$

The first part of Equation (A2) is positive when sodium channels are open, that is *g*_Na_ > 0. This is interpreted by the neuron as depolarization or excitation. In the presence of leakage currents the simple neuron model will need an additional boost to maintain the term $C\frac{dV}{dt}$ positive (or non-negative) to counteract the capacitor and the leak current. This boost can be provided by applying synaptic stimulation as in the case of the SNc dopamine model neuron.
